# The cytokine alterations/abnormalities and oxidative damage in the pancreas during hypertension development

**DOI:** 10.1007/s00424-019-02312-0

**Published:** 2019-10-17

**Authors:** Anna Kozłowska, Paweł Wojtacha, Michał Majewski, Maciej Równiak

**Affiliations:** 1 Department of Human Physiology, School of Medicine, Collegium Medicum, University of Warmia and Mazury, Olsztyn, Poland; 2 Department of Industrial and Food Microbiology, Faculty of Food Science, University of Warmia and Mazury, Olsztyn, Poland; 3 Department of Pharmacology and Toxicology, School of Medicine, Collegium Medicum, University of Warmia and Mazury, Olsztyn, Poland; 4 Department of Animal Anatomy and Physiology, Faculty of Biology and Biotechnology, University of Warmia and Mazury, Olsztyn, Poland

**Keywords:** Pancreas, Hypertension, SHR rats, Cytokines, Chemokines, Oxidative stress markers

## Abstract

The aim of the present study was to compare the content of cytokines, chemokines, and oxidative stress markers in the pancreas of spontaneously hypertensive rats (SHRs) and Wistar Kyoto Rats (WKYs) serving as controls. Enzyme-like immunosorbent assay (ELISA) and biochemical methods were used to measure pancreatic levels of interleukin-1ß, interleukin-6, tumor necrosis factor α, transforming growth factor β, RANES, monocyte chemoattractant protein 1, interferon gamma-induced protein 10, malondialdehyde, and sulfhydryl groups. The results showed that the pancreatic concentrations of all studied cytokines and chemokines did not differ between 5-week-old SHRs and WKYs, except RANTES which was significantly reduced in juvenile SHRs. In 10-week-old animals, except interleukin-1ß, the levels of all these proteins were significantly reduced in SHRs. The pancreatic levels of malondialdehyde were significantly reduced in 5-week-old SHRs and significantly elevated in 10-week-old SHRs while the contents of sulfhydryl groups were similar in both rat strains at any age studied. In conclusion, these data provide evidence that in maturating SHRs, the pancreatic levels of cytokines and chemokines are significantly reduced, while malondialdehyde significantly elevated. This suggests that in the pancreas of mature SHRs, the inflammation process is suppressed but there is ongoing oxidative damage.

## Introduction

It is generally known that the pancreas is an exocrine and endocrine organ. These functions are performed by acinar cells responsible for secretion of the pancreatic juice containing various digestive enzymes and endocrine cells responsible for release of pancreatic hormones such as glucagon (α cells); insulin, amylin, and C-peptide (β cells); pancreatic polypeptide (γ cells); somatostatin (δ cells); and ghrelin (ɛ cells) [7, 13, 33, 72]. It should be kept in mind, however, that under physiological conditions there are many external factors affecting exocrine and/or endocrine pancreatic secretion such as some hypothalamic neuropeptides (ghrelin, orexin A and B), cholecystokinin, serotonin and/or melatonin [13].

Pancreatic hormones play especially an important role in the regulation of glucose homeostasis [61]. Thus, dysfunctions of this organ usually lead to diabetes mellitus. On the other hand, diabetes mellitus may change the morphology and functions of the pancreas and can lead to other serious disorders [53]. For example, it was reported that type 1 and type 2 diabetes usually lead to the reduction of the pancreatic volume [44, 47]. Moreover, irregular pancreatic morphology associated with the decrease in the number of insulin-producing β cells was also observed in diabetic patients [9, 17, 37, 49]. In both types of diabetes, a deficit of β cell mass leads to insulin deficiency and hyperglycemia [27, 66]. It is worth mentioning that diabetes might be associated with chronic pancreatitis as well as pancreatic cancer [25**–**26]. It was also found that the inflammatory processes alone are highly involved in pancreatic cancer pathogenesis [28]. For pancreatic cancer, a recent study showed that hypertension can also increase the risk of this disease [39]. Moreover, many years ago, a relationship between acute pancreatitis and malignant hypertension with renal failure was also demonstrated [5].

Hypertension is one of the most common causes of mortality in both, developed and developing countries. As hypertension is a very serious social problem, several experimental animal models were developed as a valuable tool to study the etiology, pathophysiology, and treatment of this disease [39]. One of these models, which spontaneously develops hypertension without any involvement of pharmacological and/or surgical methods, is the genetic strain of hypertensive rat known as the spontaneously hypertensive rat (SHR) [52]. Moreover, SHR is not only a model of hypertension but it also displays various consequences associated with this condition such as cardiac hypertrophy, cardiac failure, and renal dysfunction [38].

Our previous work with the use of SHR model to study selected factors responsible for pathogenesis of ADHD [36] revealed that the levels of various cytokines (interleukin-1β: IL-1β, IL-6, tumor necrosis factor alfa and transforming growth factor beta), chemokines (regulated on activation, normal T cell expressed and secreted, monocyte chemoattractant protein-1, and interferon gamma-induced protein 10), and oxidative stress markers (malondialdehyde and sulfhydryl group) in the serum and/or spleen were significantly elevated in 5-week-old SHR rats (SHRs) when compared to age-matched control strain (Wistar Kyoto Rats, WKYs). However, to the best of our knowledge, there is no data available regarding the pancreatic content of these substances in the juvenile (5-week-old) and maturating (10-week-old) SHRs and WKYs. Such data seems to be important because it was earlier reported that an adult SHR develops spontaneous pancreatitis [54]. Moreover, it develops also hypertension [45], and recent evidence clearly demonstrated that sustained hypertension increases pancreatic oxidative stress which might lead to the pancreas damage in the hypertensive rats [23]. Thus, it seems that elevated levels of cytokines, chemokines, and oxidative stress markers observed in the serum and/or spleen of juvenile SHRs might be involved in development of pancreatitis in the maturating animals [36]. To test this hypothesis, enzyme-linked immunosorbent assay was used to detect the pancreatic levels of cytokines and chemokines. Furthermore, biochemical methods were used to investigate the oxidative stress markers in this organ.

## Materials and methods

### Animals

Juvenile (5-week-old) and maturating (10-week-old) male spontaneously hypertensive rats (SHRs, *n* = 12) and Wistar Kyoto Rats (WKYs, *n* = 12) were used in the present study. Both these time points of the rat’s lifetime were intentionally chosen. Considering that pre-pubertal SHRs are characterized primarily by ADHD abnormalities and symptoms [30], and they are devoid of hypertension [57], 5-week-old animals were selected for investigation. In post-pubertal and mature SHRs, ADHD symptoms disappear [30] but hypertension develops [57]; thus, 10-week-old animals were chosen. Both SHRs and WKYs aged 3-week were provided by Charles River (Germany). All subjects were housed in groups of two or three in sanitized polypropylene cages (to prevent isolation stress) under controlled temperature (21 ± 1 °C), 12/12-h light/dark cycle (lights on 06:00 to 18:00) and ventilated (12–20 exchanges/h) animal room. All animals were fed with a grain mixture (VRF1 diet; Charles River, Germany) and tap water ad libitum. All experiments were carried out in accordance with the European Union Directive for animal experiments (2010/63/EU) and approved by the Local Ethical Commission of the University of Warmia and Mazury in Olsztyn (no. 43/2014). All efforts were made to minimize animal suffering and to use the minimum number of animals necessary to produce reliable scientific data.

### Experimental procedure

Following the habituation phase, the experimental rats were divided into four groups according to study design: (1) 5-week-old SHR rats (*n* = 6; b.w. 111.1–123.38 g); (2) 5-week-old WKY rats (*n* = 6; b.w. 111.25–130.96 g); (3) 10-week-old SHR rats (*n* = 6; b.w. 254.72–281.38 g), and (4) 10-week-old WKY rats (*n* = 6; b.w. 247.33–266.95 g).

### Pancreas collections

Rats were deeply anesthetized with an intraperitoneal injection of Morbital (Biowet, Poland; 50 mg/kg); then, the pancreases were carefully dissected from all studied animals. All these tissue samples were immediately placed in liquid nitrogen (− 196 °C) for 30 min and then stored at low temperature (− 80 °C) for further analyses.

### Immunoenzymatic determination (ELISA) of cytokines, chemokines, and oxidative stress biomarkers in the pancreas

To determine concentrations of cytokines, chemokines, and oxidative stress markers in the rat tissues, commercial ELISA Kits were used according to the manufacturer’s instructions (Table 1). The absorbance in ELISA test plate was measured by plate reader TECAN infinite m200 pro (Austria) at the wavelength *λ* = 492 nm.

**Table 1:** The ELISA kits used for the determination of cytokine and chemokine concentrations in the present study

	Antigen	ELISA kit catalogue number	Manufacturer, country	Assay range (pg/ml)
1.	RAT IL-1β	Rat IL-1β Mini ABTS ELISA Development Kit 900-M91	Peprotech, USA	63–4000 pg/ml Intra-assay: CV < 9% Inter-assay: CV < 10%
2.	RAT IL-6	Rat IL-6 Mini ABTS ELISA Development Kit 900-M86	Peprotech, USA	31–2000 pg/ml Intra-assay: CV < 9% Inter-assay: CV < 10%
3.	RAT TNF-α	Rat TNF-α Mini TMB ELISA Development Kit 900-TM73	Peprotech, USA	47–6000 pg/ml Intra-assay: CV < 9% Inter-assay: CV < 10%
4.	TGFβ	TGF beta-1 Multispecies Matched Antibody Pair, CHC1683	ThermoFisher Scientific, USA	62.5–4000 pg/ml Intra-assay: CV < 6% Inter-assay: CV < 5%
5.	RAT MCP-1	Rat MCP-1 (CCL-2) Mini ABTS ELISA Development Kit 900-M59	Peprotech, USA	16–2000 pg/ml Intra-assay: CV < 9% Inter-assay: CV < 10%
6.	RAT RANTES	Rat RANTES (CCL5) Mini ABTS ELISA Development Kit 900-M72	Peprotech, USA	16–2000 pg/ml Intra-assay: CV < 9% Inter-assay: CV < 10%
7.	RAT IP-10	Rat IP-10 (CXCL10) Mini ABTS ELISA Development Kit 900-M449	Peprotech, USA	16–1000 pg/ml Intra-assay: CV < 9% Inter-assay: CV < 10%

### Measurement of malondialdehyde and sulfhydryl group in the pancreas

The level of malondialdehyde (MDA) and sulfhydryl groups (-SH) was measured according to the method described earlier by Weitner et al. [71] as well as Chan and Wasserman [10], respectively with own modifications. All details concerning both of these methods were described in our previous paper Kozłowska et al. [36].

### Statistical analysis

The Mann-Whitney *U* test was conducted for significant differences between WKYs and SHRs using GraphPad Prism 6 software (Graph Pad Software, La Jolla, CA, USA). *p* < 0.05 was considered to be statistically significant.

## Results

### The concentration of cytokines, chemokines, and oxidative stress markers in the pancreas

In the present study, the levels of almost all cytokines, chemokines, and/or oxidative stress markers (except –SH groups) differed significantly when animals from both age periods and/or strains were compared.

### Cytokines

**Fig. 1:**
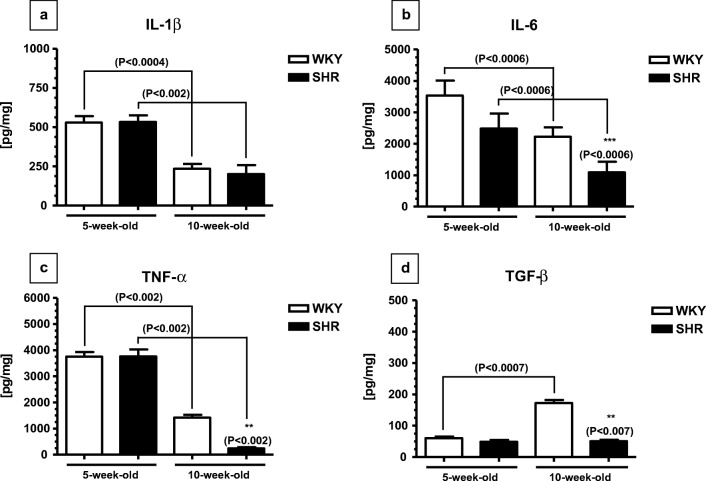
The level of IL-1ß (**a**), IL-6 (**b**), TNF-α (**c**), and TGF-β (**d**) in the pancreas of SHR (*n* = 6) and WKY rats (*n* = 6). The following statistical levels were applied: *p* < 0.05 indicates differences between the juvenile and mature rats of the same strain; **, *** indicate differences (*p* < 0.01; *p* < 0.001) between the SHR and WKY rats

The concentrations of interleukin-1ß (IL-1ß), IL-6, tumor necrosis factor α (TNF-α), and transforming growth factor β-1 (TGF-β) did not differ in 5-week-old SHRs and WKYs (Fig. 1a–d). These concentrations significantly dropped in 10-week-old animals of both rat strains (except TGF-β), but reductions were particularly strong in SHRs. In effect, the concentration of IL-6, TNF-α, and TGF-β was significantly lower in 10-week-old SHRs when compared to age-matched WKYs (Fig. 1b–d).

### Chemokines

**Fig. 2:**
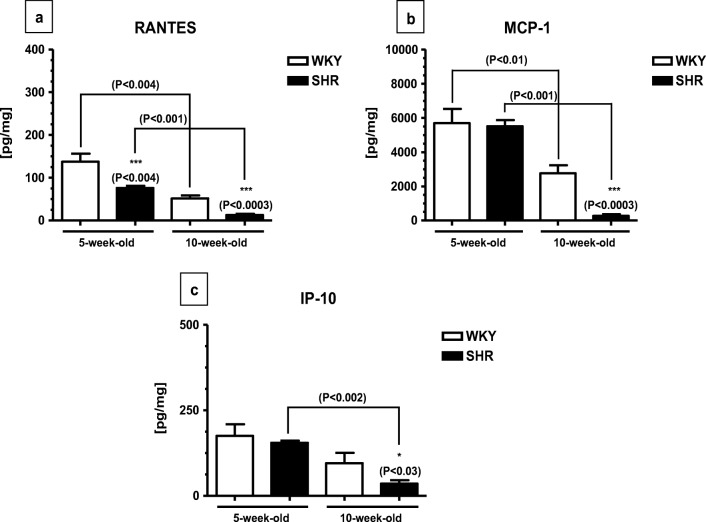
The level of RANTES (**a**), MCP-1 (**b**), and IP-10 (**c**) in the pancreas of SHR (*n* = 6) and WKY rats (*n* = 6). The following statistical levels were applied: *p* < 0.05 indicates differences between the juvenile and mature rats of the same strain; *, *** indicate differences (*p* < 0.05; *p* < 0.001) between the SHR and WKY rats

The pattern of chemokine contents was quite similar to that of cytokine contents. For example, these levels were significantly higher in 5-week-old WKYs and SHRs than in their 10-week-old counterparts (Fig. 2a–c). Furthermore, the levels of monocyte chemoattractant protein-1 (MCP-1) and interferon gamma-induced protein 10 (IP-10) did not differ in 5-week-old animals and were significantly reduced in 10-week-old SHRs (Fig. 2a–c). The level of RANTES was significantly reduced in SHRs at any age studied (Fig. 2a).

### Oxidative stress markers

**Fig. 3:**
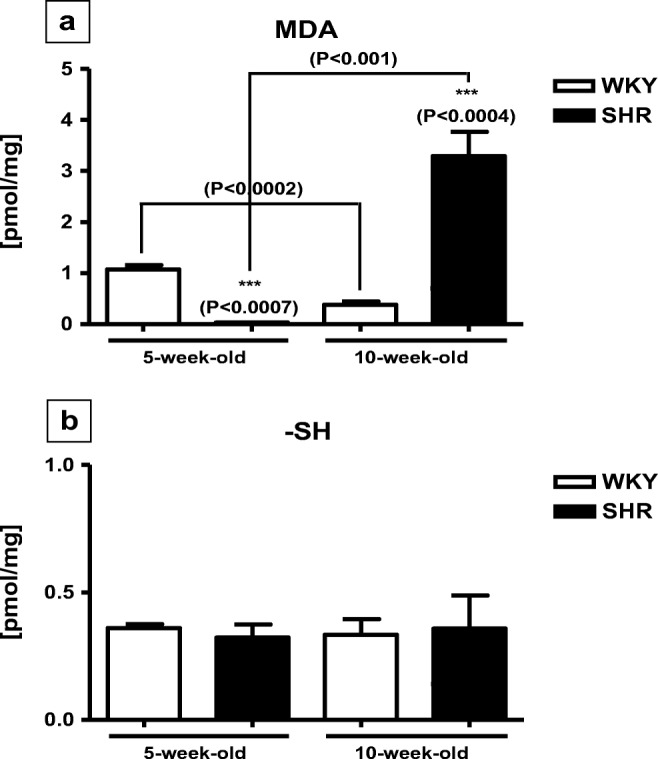
The level of MDA (**a**) and –SH (**b**) in the pancreas of SHR (*n* = 6) and WKY rats (*n* = 6). The following statistical levels were applied: *p* < 0.05 indicates differences between the juvenile and mature rats of the same strain; *** indicate differences (*p* < 0.001) between the SHR and WKY rats

The pancreatic levels of MDA were significantly reduced in 5-week-old SHRs when compared to age-matched WKYs (Fig. 3a). In contrast, in 10-week-old SHRs, these levels were significantly elevated (Fig. 3a). The concentrations of –SH did not differ between SHRs and WKYs at any of the age studied and they were quite similar in 5-week-old and 10-week-old animals (Fig. 3b).

## Discussion

This is the first paper that provides a description of selected cytokines, chemokines, and oxidative stress marker contents in the pancreas of juvenile and maturating SHRs and WKYs. The results show that the pancreatic levels of cytokines and/or chemokines did not differ in juvenile animals of both rat strains but they are significantly reduced in maturating SHRs. The pancreatic levels of MDA were significantly reduced in juvenile SHRs and significantly elevated in maturating SHRs while the content of sulfhydryl groups did not differ in both rat strains at any age studied. These results suggest that in the pancreas of maturating SHRs, the inflammation process is strongly suppressed while in parallel, a slow oxidative damage develops. However, it should be kept in mind that abnormalities observed in the present study are rather before or just at the very beginning of establishing pathological changes as various degenerative alterations in the pancreatic tissues due to spontaneous pancreatitis became evident in SHRs at the age of 12 weeks and they become more prominent or severe in older animals [54].

### Pancreatic cytokines

The present results demonstrate that the pancreatic levels of various cytokines were quite similar in 5-week-old SHRs when compared to age-matched WKYs. With age in both rat strains, these levels usually undergo significant reductions which were especially strong in SHRs. In effect, the contents of IL-6, TNF-α, and TGF-β were significantly lower in 10-week-old SHRs when compared to age-matched WKYs. These results are very interesting and quite surprising as they significantly differ from the results reporting the pattern of cytokine content in the serum and spleen in our previous study [36]. For example, in the serum and spleen, the levels of IL-1β, IL-6, and TNF-α were significantly elevated in 5-week-old SHRs when compared to age-matched WKYs whereas in 10-week-old of both rat strains, these levels were similar [36]. It is difficult to explain the reason for these differences because in the available literature, data on this topic is lacking. However, one of the possible explanations is the fact that the inflammation may show unique features in different body organs that was previously observed in the heart tissue of aged rats following severe acute pancreatitis [2]. The results of the present study demonstrate also that the pancreatic levels of IL-1β, IL-6, and TNF-α were significantly higher in 5-week-old SHRs and WKYs when compared to their 10-week-old counterparts. Thus, these results coincide well with the studies of Kiely et al. [35] who suggested that the enhanced level of various pro-inflammatory cytokines during β cell growth is probably required for their protection and survival. There is also evidence from studies in humans that there is continuous increase of β cell mass from neonates through children to reach a stable level in adolescents [65]. The present results show also that the levels of IL-6 and TNF-α were significantly reduced in 10-week-old SHRs when compared to age-matched WKYs. Although, data on the pancreatic levels of IL-6 and TNF-α in maturating SHRs is lacking, we can assume that the reduction in these pro-inflammatory cytokine content may be at least partially associated with an elevated serum and/or adrenal contents of progesterone (P_4_) and glucocorticoids (GC) observed in these animals [36–37]. Such supposition may be supported by well-known facts that P_4_ and GC might inhibit secretion of IL-6 and TNF-α [16, 24, 68]. Moreover, there is a strong dependence between TNF-α and GC causing that this cytokine might reduce 11β-hydroxysteroid dehydrogenase types 2 activity and in this way increase GC access to their receptors to modulate the inflammatory response [29]. The pattern of TGF-β content in the present study was quite different from that of IL-6 and TNF-α. For example, the level of this cytokine was significantly higher in maturating WKYs than in juvenile WKYs. The reason for this increase is not fully understood. However, there is evidence that higher level of TGF-β promotes Foxp3 expressing Treg cells which are critical in maintaining self-tolerance and immune homeostasis [69, 78]. Thus, it is plausible that in this way 10-week-old WKYs developed immune tolerance [48, 77]. On the other hand, the levels of TGF-β in SHRs did not change with age and in effect being significantly lower in 10-week-old SHRs than in 10-week-old WKYs. Significantly reduced content of TGF-β in maturating SHRs was also observed in the spleen [36]. Interestingly, low levels of TGF-β together with IL-6 and IL-21 promote IL-23 receptor expression and in this way stimulate Th17 cell differentiation (by inducing RORγt expression) [78]. As Th17 cells play an important role in variety of human autoimmune diseases, it is plausible that lowered TGF-β content in maturating SHRs may be a mark of reduced immune tolerance in these animals [31, 78].

### Pancreatic chemokines

The present results demonstrate that the pancreatic levels of RANTES, MCP-1, and IP-10 were significantly higher in the 5-week-old SHRs and WKYs than in their maturating counterparts. Furthermore, the levels of MCP-1 and IP-10 did not differ in 5-week-old animals and were significantly reduced in 10-week-old SHRs whereas the level of RANTES was significantly reduced in SHRs at any age studied. Thus, the pattern of pancreatic chemokine contents mimics that of pancreatic cytokine contents and is very different from the pattern of chemokine contents in the serum and spleen [36]. It should be pointed out that there is a lack of data concerning the pancreatic levels of RANTES, MCP-1, and IP-10 in the juvenile and/or maturating SHRs. As of yet, it was only reported that some chemokines might (similarly to cytokines) promote pancreatic β cell protection and survival during their development which could explain elevated levels of these proteins in juvenile animals [14]. Moreover, low levels of MCP-1 due to suppression by elevated amounts of P_4_ and GC were previously observed by several authors [34, 50, 76] what coincide well with lowered chemokine contents in 10-week-old SHRs (present study). Based on the present data, it can be assumed that the low levels of selected chemokines (and pro-inflammatory cytokines) found in the 10-week-old SHRs are not accidental and may play an important role in the attenuation of inflammatory process [42]. However, it should be kept in mind that cytokines and/or chemokines are required during proper pancreas development and in normal pancreatic tissue maintenance. For example, as it was already mentioned, they might promote pancreatic β cell protection and survival during development [14, 35]. These proteins also direct ductal-to-endocrine cell differentiation, with implications for β cell regeneration (via STAT3-dependent NGN3 activation) [74]. In the mature pancreas, cytokines such as IL-1β, IL-6, TNF-α, and TGF-β seem to be involved in the regulation of pancreatic chemokine, insulin, and/or glucagon secretion [4, 8, 41, 64] while chemokine MCP-1 which is constitutively present in pancreatic islet cells might play a role as a chemotactic factor [56]. The role of cytokines/chemokines in the pancreatic homeostasis may demonstrate among others studies on p38 mitogen-activated protein kinase which upregulates various cytokines and chemokines including IL-6, TNF-α, and MCP-1 [6] and at the same time suppresses chronic pancreatitis [77].

### Pancreatic oxidative stress markers

The present results revealed that the pancreatic levels of MDA were significantly lower in 5-week-old SHRs when compared to age-matched WKYs whereas in 10-week-old animals, these levels were significantly higher in SHRs than WKYs. The concentrations of –SH did not differ between SHRs and WKYs at any of the age studied. Thus, the pancreatic pattern of oxidative stress markers differs significantly from that in the spleen [36]. For example, in the pancreas, the level of MDA was significantly higher in juvenile than in maturating WKYs while in the spleen, the levels of MDA did not differ between juvenile and maturating rats of the same strain [36]. It is possible that in the pancreas of WKYs, the elevated level of TGF-β might modulate lipid peroxidation levels. Such mechanism was reported in rabbits in which during oral mucosal wound healing and after TGF-β administration, the nitric oxide and MDA levels increased on the third day to decrease on day 5 after wounding [15]. However, this assumption needs to be verified experimentally, since in the available literature, there is a lack of data addressing this topic in detail. The present results revealed also that the pancreatic level of MDA was significantly reduced in 5-week-old SHRs when compared to 5-week-old WKYs and 10-week-old SHRs. This result is also in contrast with our previous findings in the spleen where the level of MDA was significantly higher in the juvenile SHRs when compared to age-matched WKYs and 10-week-old rats of both strains [36]. We suppose that difference between pancreatic and splenic level of MDA may be a consequence of different oxidative stress levels which is organ specific [32]. In addition, strongly reduced pancreatic level of MDA in 5-week-old SHRs might be associated with internal mechanisms that protect the pancreas from oxidative damage. Such protection seems to be especially important in SHRs because in young and adult SHRs, the β cell component of pancreatic islets is reduced when compared to normotensive Wistar rats [58]. Moreover, the pancreas (especially pancreatic β cells) is sensitive to oxidative stress and pancreatic β cells had lower levels of antioxidative enzymes when compared to the liver [67, 70]. In turn, strong elevation of pancreatic MDA level in 10-week-old SHRs, observed in the present study, could be associated with progressive oxidative damage of this organ which may lead finally to pancreatitis [40, 51]. This assumption is supported by the results in adult SHRs in which spontaneous pancreatitis was found [54]. Moreover, it was reported that low expression of the mitochondrial superoxide dismutase (SOD) results in higher concentration of MDA in the SHR brain [11]. Thus, similar situation in the pancreas cannot be ruled out. It is generally accepted that the SOD is the first line of defense against superoxide anion radical (O2¯*) because it catalyzes dismutation of O2¯* to hydrogen peroxide [3]. It is plausible that an increase of O2¯* in SHRs is connected with depletion of the SOD which could lead to peroxidation of lipids and in consequence to the higher level of MDA likewise [12]. However, further studies are necessary. It is worth mentioning here that although elevated levels of MDA in mature SHRs indicate an ongoing oxidative damage of the pancreas, this aldehyde is only the main product of lipid peroxidation [51] and data on other oxidative mechanisms in this organ is still lacking. Protein oxidation, i.e., methionine residue oxidation, tyrosine, or tryptophan residue oxidation could also shed some light in the future on the pancreatic pathology in mature SHRs. The present results indicated also that the pancreatic contents of sulfhydryl groups did not differ in both rat strains at any age studied. This phenomenon may be explained by the fact that –SH group is closely related with the level of glutathione (GSH) which in cells is the basic antioxidative substance [19]. GSH can react with sulfenic acid and reduce to –SH group [43] which is formed from this group during oxidative stress [46]. Interestingly, TNF-α is one of the most important agents to activate synthesis of GSH [59], and in the present study, the level of TNF-α in maturating SHRs is reduced. On the other hand, low concentration of –SH group observed in the present study might also be due to the high level of GC [36], which causes depletion of GSH and activity decrease of γ-glutamylcysteine synthetase what was earlier observed in the alveolar epithelial cells after dexamethasone (synthetic GC) administration [59].

The present results provide evidence that in maturating SHRs, the pancreatic levels of cytokines and chemokines are significantly reduced, while malondialdehyde significantly elevated. This suggests that in the pancreas of mature SHRs, the inflammation process is suppressed but there is ongoing oxidative damage. This may also suggest that in mature SHRs, inflammation is rather inversely correlated with oxidative stress. Generally less inflammation should be correlated to less oxidative stress and such phenomenon is clearly visible in mature WKYs. However, SHRs during lifetime develop ADHD [63] and hypertension [57], and they have significantly altered serum [37] and adrenal [36] concentrations of various steroid hormones which have direct influence on cytokine/chemokine synthesis and oxidative stress. For example, it is widely accepted that P_4_ and GC might downregulate a great number of cytokines such as IL-1β, IL-6, IL-8, IL-12, IL-18, and TNF-α as well as chemokines, such as RANTES and MCP-1 [16, 18, 20–22, 24, 60, 68, 73]. On the other hand, GC might increase oxidative stress [1]. Thus, steroid hormone upregulation in mature SHRs seems to be enough potent factor to downregulate cytokine/chemokine synthesis on the one hand and to increase oxidative stress on the other. Another potent factor which may have huge impact on oxidative stress in mature SHRs is hypertension. For example, recent evidence clearly demonstrated that sustained hypertension increases pancreatic oxidative stress which might lead to the pancreas damage in the hypertensive rats [23]. It is worth mentioning that an anti-inflammatory and protective mechanism in maturing SHRs through steroid hormone upregulation coincides with studies in WBN/Kob rats which are another animal model of chronic pancreatitis. For example, in male WBN/Kob rats, TNF-α and IL-6 concentrations peak well before the peak of disease severity what may suggest that both these proteins are involved in the onset of pancreatitis [75]. However, estrogen-treated males and non-treated females (with healthy ovaries producing estrogens and/or P_4_) do not develop pancreas damage suggesting that female sex hormones may be quite efficient protecting mechanism [62]. Steroid hormone levels including P_4_ and GC are unfortunately unknown in WBN/Kob rats and these animals do not develop hypertension. In maturing male SHRs, P_4_ and GC are highly elevated but estradiol is not [37], and these rats develop hypertension [57]. Thus, it seems that in SHRs, steroid hormone upregulation is sufficient to treat inflammation but it may be insufficient to counteract pancreas damage.

In conclusion, the present study provides evidence that the pancreatic levels of cytokines and/or chemokines are significantly reduced, while MDA significantly elevated in the maturating SHRs when compared to age-matched WKYs. This suggests that in the pancreas of maturating SHRs, the inflammation process is suppressed while in parallel, a slow oxidative damage develops. Moreover, a comparison of the present results with our previous studies [36–37] suggests that both these processes in mature SHRs could be induced by highly elevated levels of steroid hormones which are enough potent to downregulate cytokine/chemokine synthesis and increase oxidative stress.

## Abbreviations

ELISA: enzyme-like immunosorbent assay
GC: glucocorticoids
GSH: glutathione
IL-1ß: interleukin-1ß
IL-6: interleukin-6
IL-21: interleukin-21
IL-23: interleukin-23
IP-10: interferon gamma-induced protein 10
MCP-1: chemoattractant protein-1
MDA: malondialdehyde
O2¯*: superoxide anion radical
P_4_: progesterone
RANTES: regulated upon activation, normal T cell expressed and secreted
-SH: sulfhydryl groups
SHR: spontaneously hypertensive rat
SOD: superoxide dismutase
TGF-β: transforming growth factor β-1
TNF-α: tumor necrosis factor α
WKY: Wistar Kyoto Rat
